# Endocardial Anchoring Technique for Variant Supracardiac Total Anomalous Pulmonary Venous Return

**DOI:** 10.1016/j.atssr.2024.01.001

**Published:** 2024-02-02

**Authors:** Yusuke Yamamoto, Sho Akiyama, Mio Noma, Shohei Senoo, Jun Maeda, Yukihiro Yoshimura

**Affiliations:** 1 Department of Pediatric Cardiovascular Surgery, Tokyo Metropolitan Children's Medical Center, Tokyo, Japan; 2 Department of Pediatric Cardiology, Tokyo Metropolitan Children's Medical Center, Tokyo, Japan

## Abstract

The endocardial anchoring technique is a novel modification of total anomalous pulmonary venous return repair that involves creation of an L-shaped flap of the pulmonary venous confluence, subsequently anchoring it to the endocardium. A wide and smooth pathway can be expected from the theoretical advantages of this technique, namely, a smooth inner surface of the anchored flap and traction force to extend the orifice of the connection. An application of this technique for a rare variant of supracardiac total anomalous pulmonary venous return suggests its potential to be an alternative to the conventional repair, especially in patients with a curved pulmonary venous confluence.

As described in our previous report,^1^ the endocardial anchoring technique was originally devised as a modification of the cutback method for cardiac total anomalous pulmonary venous return (TAPVR), aiming to provide a wide and smooth pathway for pulmonary venous drainage. The technique consists of 2 maneuvers: making a flap by an L-shaped incision at the pulmonary venous confluence and anchoring the reversed flap to the endocardium of the left atrium. The simple and adaptive characteristics of this technique allow its extended application to other types of TAPVR. Herein, we describe a patient with an atypical variant of supracardiac TAPVR who was successfully treated by this technique.

A female neonate weighing 1.9 kg was born at 38 weeks’ gestation with a diagnosis of double-outlet right ventricle, hypoplastic left ventricle, mitral atresia, ventricular septal defect, atrial septal defect, and atypical variant of supracardiac TAPVR. Computed tomography imaging revealed a perpendicularly curved common pulmonary venous confluence, which consisted of all 4 pulmonary veins, draining into 2 discrete pathways: the vertical vein connecting to the innominate vein and a small tubular connection to the left atrium (Figure 1). As a Fontan candidate, the patient underwent pulmonary artery banding and enlargement of the atrial septal defect on postnatal day 9, which was to be followed by later adjustment of the pulmonary artery band in accordance with somatic growth. Although the Glenn procedure was planned as a second-stage palliation, progressive obstruction of both the vertical vein and the tubular connection developed during the interstage period, which was prolonged because of repetitive respiratory infections in the patient. Considering the elevated pulmonary vascular resistance, surgical repair of TAPVR before the Glenn procedure was indicated at 10 months of age with a body weight of 5.5 kg.

**Figure 1:**
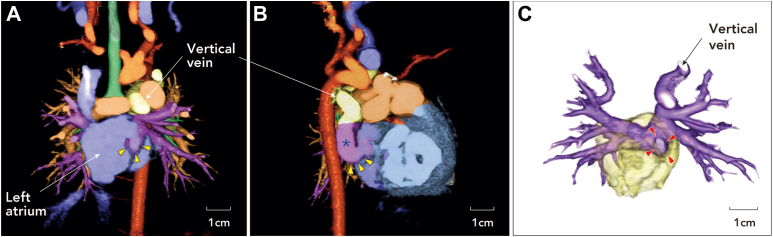
Preoperative 3-dimensional volume rendering of computed tomography images taken at the age of 5 months, before the appearance of the pulmonary venous obstruction. (A) Coronal image and (B) sagittal image showing the tubular connection (arrowheads) between the pulmonary venous confluence (asterisk) and the left atrium. (C) The entire image of the pulmonary venous system viewed from the front, with the posterior wall of the left atrium drawn in transparent yellow. The orifice of the connection is indicated by arrowheads.

## Technique

**Figure 2:**
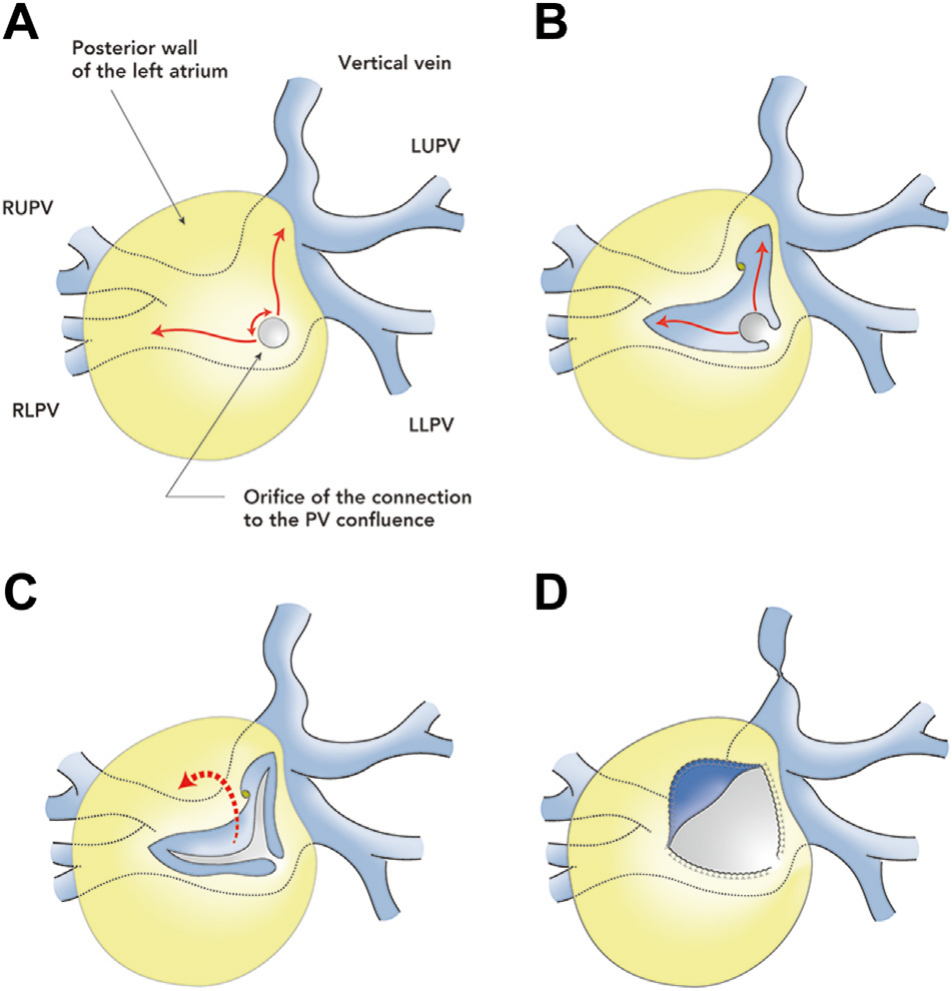
Schematic view of the endocardial anchoring technique for an atypical variant of supracardiac total anomalous pulmonary venous return. (A) The posterior wall of the left atrium viewed from inside (yellow area) is incised in an upward and rightward direction from the orifice of the tubular connection to the pulmonary venous (PV) confluence. (B) Through the triangular opening in the posterior wall of the left atrium, the anterior aspect of the pulmonary venous confluence is incised in an upward and rightward direction. (C) The L-shaped flap created at the pulmonary venous confluence is drawn into the atrial cavity. (D) The L-shaped flap is anchored to the endocardium of the left atrium with a running suture. The remaining 2 incision lines of the pulmonary venous confluence are also sutured with their respective counterparts on the left atrium. (LLPV, left lower pulmonary vein; LUPV, left upper pulmonary vein; RLPV, right lower pulmonary vein; RUPV, right upper pulmonary vein.)

**Figure 3:**
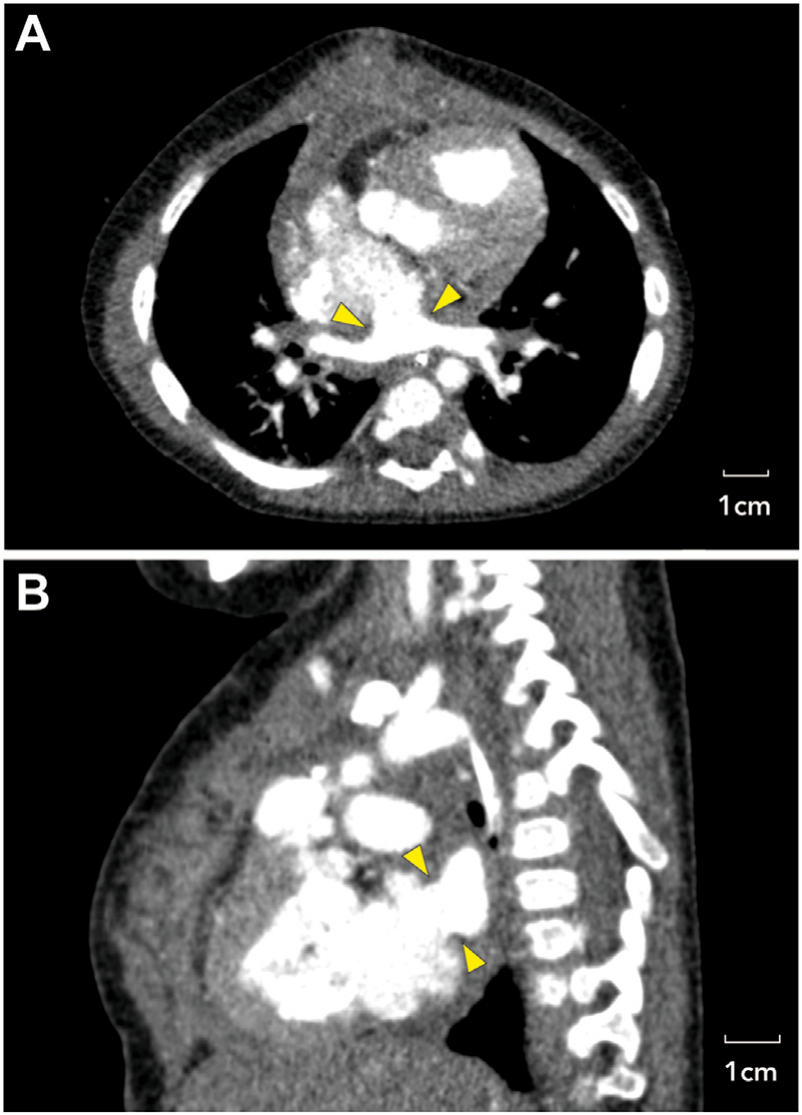
Computed tomography images taken 1 month postoperatively. (A) Axial image and (B) sagittal image showing widely patent connection between the pulmonary venous confluence and the left atrium (arrowheads).

Cardiopulmonary bypass was established with aortobicaval cannulation through median sternotomy. The vertical vein and tubular connection to the left atrium were exposed circumferentially through careful dissection of the pulmonary venous confluence. After cardioplegic arrest, the posterior wall of the left atrium was incised, through the previously enlarged atrial septal defect, in an upward and rightward direction from the orifice of the tubular connection. Through this window, the anterior aspect of the pulmonary venous confluence was incised upward to the point just below the orifice of the left upper pulmonary vein and rightward to the midpoint of the orifices of the right pulmonary veins. The L-shaped flap created at the pulmonary venous confluence was then drawn into the atrial cavity and anchored to the endocardium of the left atrium with a running suture, applying an adequate traction force onto the flap. The remaining 2 incision lines of the pulmonary venous confluence were sutured with their respective counterparts on the left atrium, with care taken to bury the irregular atrial muscle of the cut edge under the suture line (Figure 2). The patient was uneventfully weaned off of cardiopulmonary bypass after readjustment of the pulmonary artery band. Computed tomography imaging at 1 month postoperatively showed a widely opened connection between the pulmonary venous confluence and the left atrium (Figure 3). Postoperatively, no anticoagulant or antiplatelet agents were administered.

## Comment

The endocardial anchoring technique is a novel maneuver for TAPVR repair that consists of making an L-shaped incision at the pulmonary venous confluence and anchoring the reversed flap to the endocardium of the left atrium. The theoretical advantages of this technique are as follows. First, the seamless inner surface of the reversed flap might contribute to a smooth channel for pulmonary venous drainage, preventing turbulent blood flow and subsequent intimal hyperplasia. Based on this hydrodynamic property, it is conceivable that any anticoagulant or antiplatelet agents are unnecessary for the postoperative management of this surgical technique. Second, the traction force produced by anchoring of the flap might lead to widening of the orifice of the connection between the pulmonary venous confluence and the left atrium. In addition, adjustment of the angle and length of the L-shaped incision might allow its application to a bent pulmonary venous confluence, wherein a sufficient length of linear incision is not available. On the other hand, there are 2 conditions in which application of this technique might be difficult: preexisting peripheral pulmonary venous obstruction that cannot be dealt with by this technique; and a restrictive interatrial connection preventing a surgical approach to the pulmonary venous confluence.

In conclusion, this technique might serve as an alternative to conventional repair, especially for TAPVR with a curved pulmonary venous confluence, provided an approach through the atrial septal defect is feasible and peripheral pulmonary venous obstruction is not present. Further follow-up is necessary to reveal the long-term outcome of this technique.
